# Identification of KLF6/PSGs and NPY-Related USF2/CEACAM Transcriptional Regulatory Networks via Spinal Cord Bulk and Single-Cell RNA-Seq Analysis

**DOI:** 10.1155/2021/2826609

**Published:** 2021-11-29

**Authors:** Xinbing Liu, Wei Gao, Wei Liu

**Affiliations:** Department of Neurosurgery, Changxing People's Hospital, Changxing, Zhejiang Province, China

## Abstract

**Background:**

To further understand the development of the spinal cord, an exploration of the patterns and transcriptional features of spinal cord development in newborn mice at the cellular transcriptome level was carried out.

**Methods:**

The mouse single-cell sequencing (scRNA-seq) dataset was downloaded from the GSE108788 dataset. Single-cell RNA-Seq (scRNA-Seq) was conducted on cervical and lumbar spinal V2a interneurons from 2 P0 neonates. Single-cell analysis using the Seurat package was completed, and marker mRNAs were identified for each cluster. Then, pseudotemporal analysis was used to analyze the transcription changes of marker mRNAs in different clusters over time. Finally, the functions of these marker mRNAs were assessed by enrichment analysis and protein-protein interaction (PPI) networks. A transcriptional regulatory network was then constructed using the TRRUST dataset.

**Results:**

A total of 949 cells were screened. Single-cell analysis was conducted based on marker mRNAs of each cluster, which revealed the heterogeneity of neonatal mouse spinal cord neuronal cells. Functional analysis of pseudotemporal trajectory-related marker mRNAs suggested that pregnancy-specific glycoproteins (PSGs) and carcinoembryonic antigen cell adhesion molecules (CEACAMs) were the core mRNAs in cluster 3. GSVA analysis then demonstrated that the different clusters had differences in pathway activity. By constructing a transcriptional regulatory network, USF2 was identified to be a transcriptional regulator of CEACAM1 and CEACAM5, while KLF6 was identified to be a transcriptional regulator of PSG3 and PSG5. This conclusion was then validated using the Genotype-Tissue Expression (GTEx) spinal cord transcriptome dataset.

**Conclusions:**

This study completed an integrated analysis of a single-cell dataset with the utilization of marker mRNAs. USF2/CEACAM1&5 and KLF6/PSG3&5 transcriptional regulatory networks were identified by spinal cord single-cell analysis.

## 1. Introduction

The nervous system is composed of the central nervous system (CNS) and the peripheral nervous system (PNS). Included in the central nervous system is the spinal cord, which plays a vital role in our perception of the environment and its interactions. The spinal cord is composed of gray matter and white matter. In the center of the spinal cord, there is a thin lumen called the central canal surrounded by the ventricular epithelium. The ventricular epithelium is then surrounded by an H-shaped gray matter followed by the white matter. The gray matter is composed of two parts, the anterior horn and the posterior horn. The anterior part of the gray matter is the anterior horn, which is larger compared to the posterior horn. There are numerous multipolar motor neurons within the anterior horn, while neurons within the posterior horn form synaptic connections with sensory ganglion cells. These neurons form a neural circuit that utilizes sensory input and converts the commands from the brain into the body's response to the environment, such as muscle contractions [1].

The discovery of the developmental trajectory of the central nervous system facilitated the exploration of the pathological process of neuronal repair; the pathogenesis of central nervous system injures and neurodegenerative diseases set the foundation for the translation of clinical treatment [1, 2]. In the early stages of mouse embryonic development, the central nervous system first develops in the ectoderm. Thereafter, stem cells differentiate into a variety of different cell types. However, the exact processes and steps involved in the differentiation of these cells are still controversial and have yet to be further investigated. These controversies revolve around whether neural development is induced or transformed [3, 4]. Recent studies now include the single-cell transcriptome that provides a way to precisely map the transcriptional heterogeneity of cells across time [5, 6]. As a result, CNS research has made tremendous progress in recent years due to the rapid advances in single-cell histology techniques [7].

This study is aimed at exploring the patterns and transcriptional features of spinal cord development in newborn mice at the single-cell transcriptome level, thereby furthering our current understanding of the developmental processes of the spinal cord and its underlying mechanisms. This understanding will allow the further investigation of the developmental trajectory of mouse neurons and the cell biology of this disease.

## 2. Materials and Methods

### 2.1. Data Collection and Computational Analysis of scRNA-Seq Datasets

The mouse scRNA-seq dataset was downloaded from the GEO database (GSE108788) [8]. The data from the scRNA-seq dataset was collected from neonatal mouse cervical and lumbar spine interneurons, which were sorted via flow cytometry. Quality control was carried out as described previously [8]. Similar to previous studies, the single-cell analysis was completed using the Seurat package [5, 9]. Briefly, the “Find neighbors” and “Find Clusters” functions from R software were utilized to perform principal component analysis and cell clustering [10]. The marker mRNAs between each cluster were identified using the FindAllMarkers function. Then, based on these marker mRNAs, the UMAP method was used to perform nonlinear dimensionality reduction. Initial annotation of the cells was done using the singleR package [11]. A pseudotemporal analysis was then performed using the Monocle 3 package, and the differences in expression of marker mRNAs on pseudotemporal trajectories were obtained for each cluster.

### 2.2. Protein-Protein Interaction (PPI) Network Analysis

The initial analysis of the PPI network was done by the STRING v11 database (http://string-db.org) (feasibility of 0.40, medium confidence level). Then, based on the obtained results, the PPI network was constructed using the R software [12].

### 2.3. Enrichment Analysis

As described in the previous study, the clusterProfiler package of the R software was utilized to complete the GO and KEGG enrichment analysis of the marker mRNAs in each cluster [13, 14]. Pathway enrichment analysis was performed with ReactomePA to identify gene sets from the Reactome database with a false discovery rate of <0.05 in the marker sets [15, 16].

### 2.4. Prediction of Transcription Factors

The TRRUST database, which contains data on the regulatory relationship between 800 human transcription factors and 828 mouse transcription factors, was used for transcription factor prediction analysis. All TF-target gene pairs included were experimentally validated [17].

### 2.5. Statistical Analysis

Data analysis and plotting of the results were done using R software (version 4.0.2). The results of the intersection analysis were visualized through a Venn diagram. An unpaired *t*-test was conducted to distinguish differences between two groups, and a *p* value of <0.05 was defined as a significant difference.

## 3. Results

### 3.1. Quality Control and Cluster Analysis of the GSE108788 Dataset

**Figure 1:**
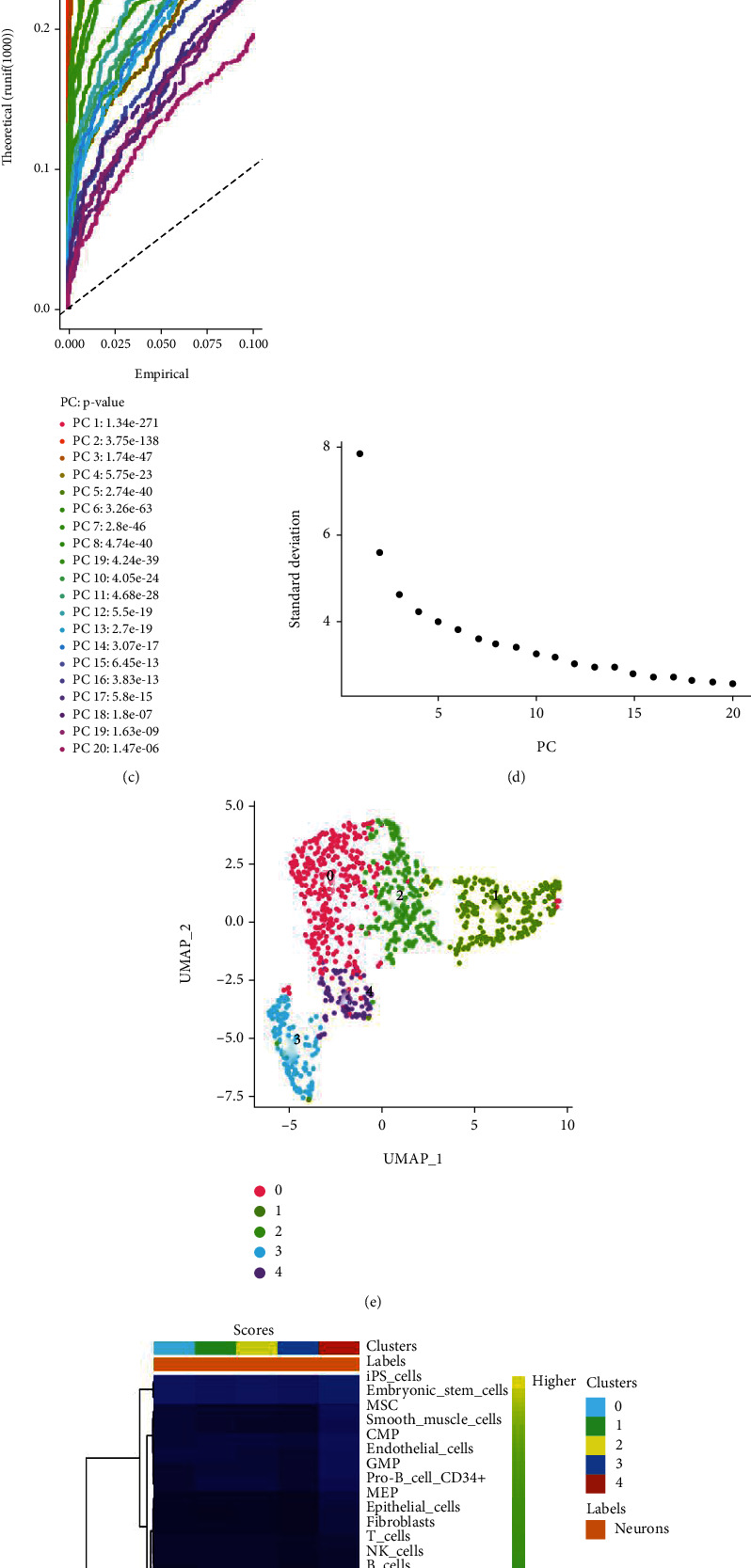
Quality control and clustering of the GSE108788 dataset. (a) Quality control process for the GSE108788 dataset. (b) ANOVA plot showing 2000 highly variable genes in mouse neuronal cells. Red indicates highly variable mRNAs; black indicates nonvariable mRNAs. (c, d) PCA analysis identified 20 clusters with *p* values less than 0.001. (e) The UMAP diagram shows the distribution of nerve cells into five clusters. (f) The singleR package was used to annotate the clusters, confirming that all of them belonged to neural cells.

To analyze the transcriptional characteristics of neonatal mouse neuronal cells at a single-cell level, we filtered the dataset GSE108788 and implemented quality control using the Seurat package for R software (Figure 1(a)). The selected 949 mouse neuronal cells were normalized using the Seurat package. Then, an analysis of variance (ANOVA) plot showed 2000 highly variable mRNAs (Figure 1(b)). PCA analysis was first used to screen 20 clusters with *p* values less than 0.001 (Figures 1(c) and 1(d)). UMAP analysis was then conducted, where five clusters were identified for further screening (Figure 1(e)). Ultimately, the singleR package was used to annotate these five clusters, confirming that all these cells were neural cells (Figure 1(f)).

### 3.2. Single-Cell Analysis Reveals the Heterogeneity of Neural Cells in the Neonatal Mouse Spinal Cord

**Figure 2:**
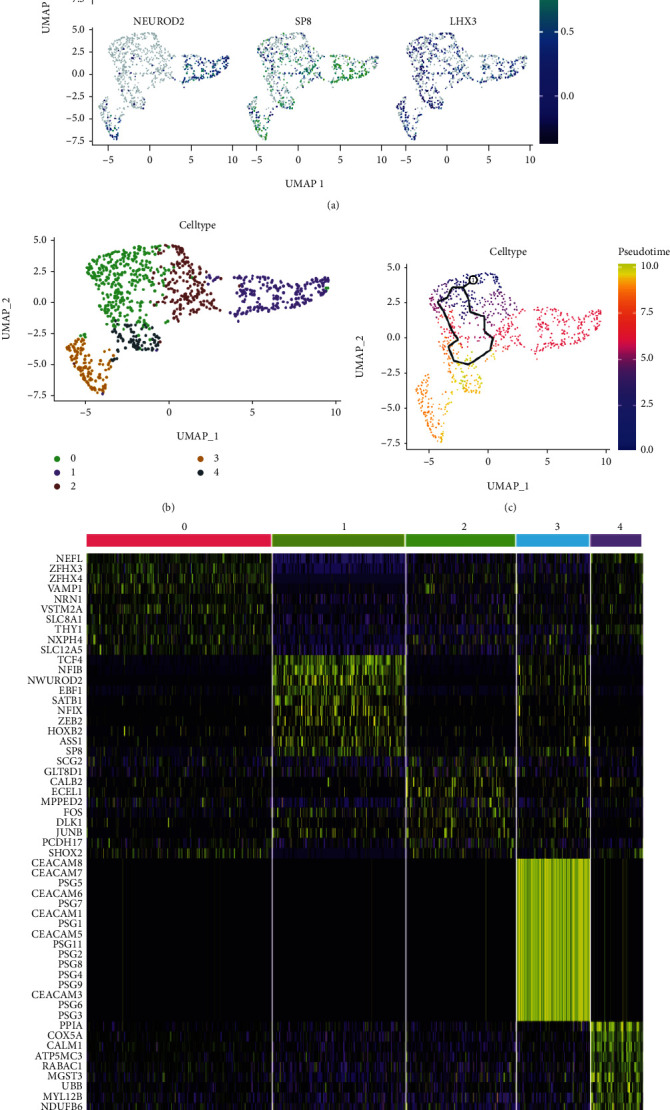
Single-cell analysis was used to reveal the heterogeneity of neonatal mouse spinal cord neuronal cells. (a) UMAP plots were used to demonstrate the expression of the markers NFIB, ZFHX3, SHOX2, NEUROD2, SP3, and LHX3. (b) These cells were divided into five cell types by PCA and UMAP analysis. (c) The top ten marker mRNAs for each cell population are shown in the heat map. (d) The proposed chronological analysis of spinal cord neuronal cells from these newborn mice.

The data from the scRNA-seq dataset was collected from neonatal mouse cervical and lumbar spine interneurons, which were sorted via flow cytometry. Based on data from previous studies, a UMAP plot was created to show the expression of the following markers: NFIB, ZFHX3, SHOX2, NEUROD2, SP8, and LHX3 (Figure 2(a)) [8]. In this study, 949 cells were screened by Seurat package for R software and were grouped into 5 clusters (Figure 2(b)). Among these cells, *ZFHX3* and *SHOX2* were jointly enriched in clusters 0, 2, and 4, while *NFIB*, *NEUROD2*, and *SP8* were jointly enriched in clusters 1 and 3. As shown in the figure, the top 10 marker mRNAs for each cell cluster are shown in the heat map. Nefl, Zfhx3, Zfhx4, Vamp1, Nrn1, Vstm2a, Slc8a1, Thy1, Nxph4, and Slc12a5 are the marker mRNAs for cluster 0; Tcf4, Nfib, Neurod2, Ebf1, Satb1, Nfix, Zeb2, Hoxb8, Ass1, and Sp8 are the marker mRNAs for cluster 1; Scg2, Glt8d1, Calb2, Ecel1, Mpped2, Fos, Dlk1, Junb, Dlk1, Junb, Pcdh17, and Shox2 are the marker mRNAs for cluster 2; Ceacam8, Ceacam7, Psg7, Ceacam6, Psg5, Psg1, Ceacam1, Ceacam5, Psg11, Psg2, Psg8, Psg3, Psg9, Ceacam3, Psg6, and Psg3 are the marker mRNAs for cluster 3; Ppia, Cox5a, Calm1, Atp5mc3, Rabac1, Mgst3, Ubb, Myl12b, Ndufb6, and Atp6voc are the marker mRNAs for cluster 4 (Figure 2(c)). We identified the Psgs and the Ceacams as important marker mRNAs for cluster 3. In the present study, spinal cord neuronal cells from newborn mice were subjected to mimetic chronological analysis on the UMAP map (Figure 2(d)). Cluster 3 was found to be at the final stage of the proposed chronological differentiation.

### 3.3. Psgs and Ceacams Are Specifically Expressed in Cluster 3

**Figure 3:**
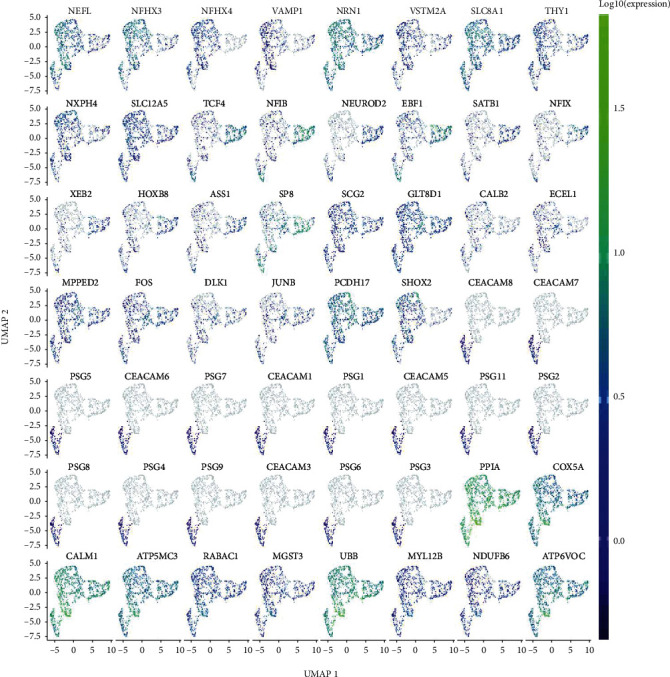
The UMAP plot of the markers in neonatal mouse spinal cord neuronal cells.

**Figure 4:**
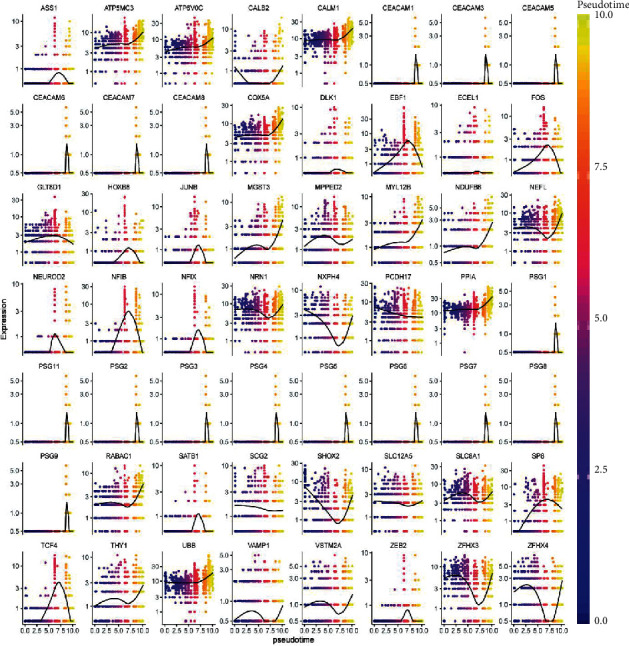
Expression of marker mRNAs varies with pseudotiming.

The UMAP plot shows the expression of all marker mRNAs in neonatal mouse spinal cord neuronal cells (Figure 3). Psgs and Ceacams were found to be specifically expressed in cluster 3. Marker mRNAs with pseudotiming analysis was shown in Figure 4. Marker mRNAs in cluster 3 (*Ceacam8*, *Ceacam7*, *Psg7*, *Ceacam6*, *Psg5*, *Psg1*, *Ceacam1*, *Ceacam5*, *Psg11*, *Psg2*, *Psg8*, *Psg3*, *Psg9*, *Ceacam3*, *Psg6*, and *Psg3*) began only at the tail end of the pseudotemporal differentiation. This observation suggests that Psgs and Ceacams may be markers of neural maturation. In addition, we also found that the expression of the genes *Atp5mc3*, *Atp6v0c*, *Calm1*, *Cox5a*, *Mgst3*, *Myl12b*, *Ndufb6*, and *Ppia* increased with increasing chronology during the process of neuronal differentiation in mice. These genes may be potential biomarkers for the degree of differentiation of mouse spinal cord neuronal cells.

### 3.4. Acquisition of Markers Related to Pseudotiming Traces

**Figure 5:**
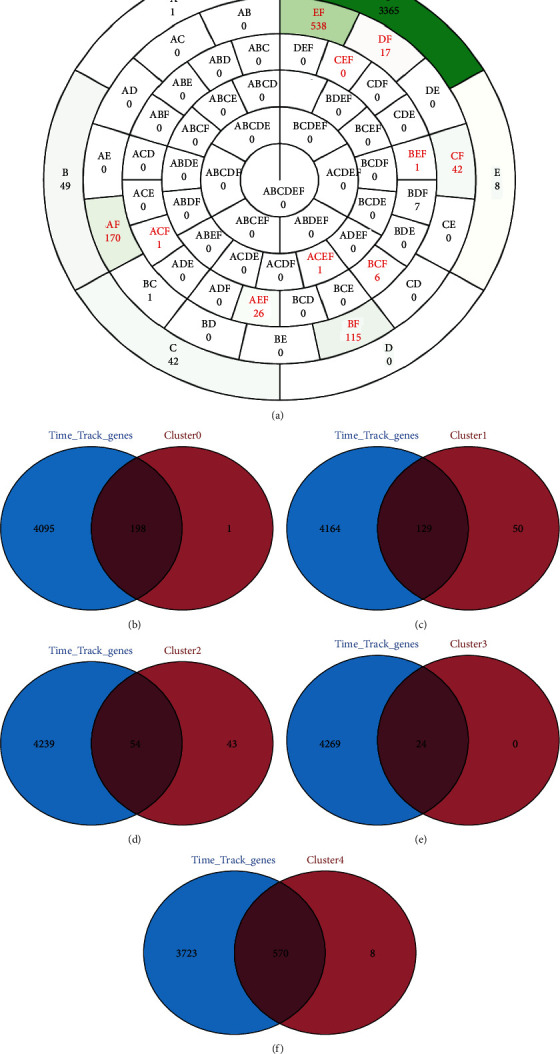
Intersection analysis of marker mRNAs and time track mRNAs for different clusters. (a) Total plot showing the intersection of different clustered marker mRNAs and time track mRNAs. Each compartment shows the cluster and the number of mRNAs variably contained within it (A: cluster 1; B: cluster 2; C: cluster 3; D: cluster 4; E: pseudotime track-related gene cluster). (b–f) Venn diagram of marker mRNAs and time track mRNAs for the different clusters.

To further elucidate the cell differentiation trajectories of various biomarkers, we conducted an intersection analysis. The general plot of the intersection analysis of marker mRNAs and time track mRNAs for the different clusters is shown in Figure 5(a). There are 198, 129, 54, 24, and 570 marker mRNAs associated with pseudotemporal trajectories in clusters 0, 1, 2, 3, and 4, respectively (Figures 5(b)–5(f)).

### 3.5. Functional Analysis of Pseudotemporal Trajectory-Related Marker mRNAs

**Figure 6:**
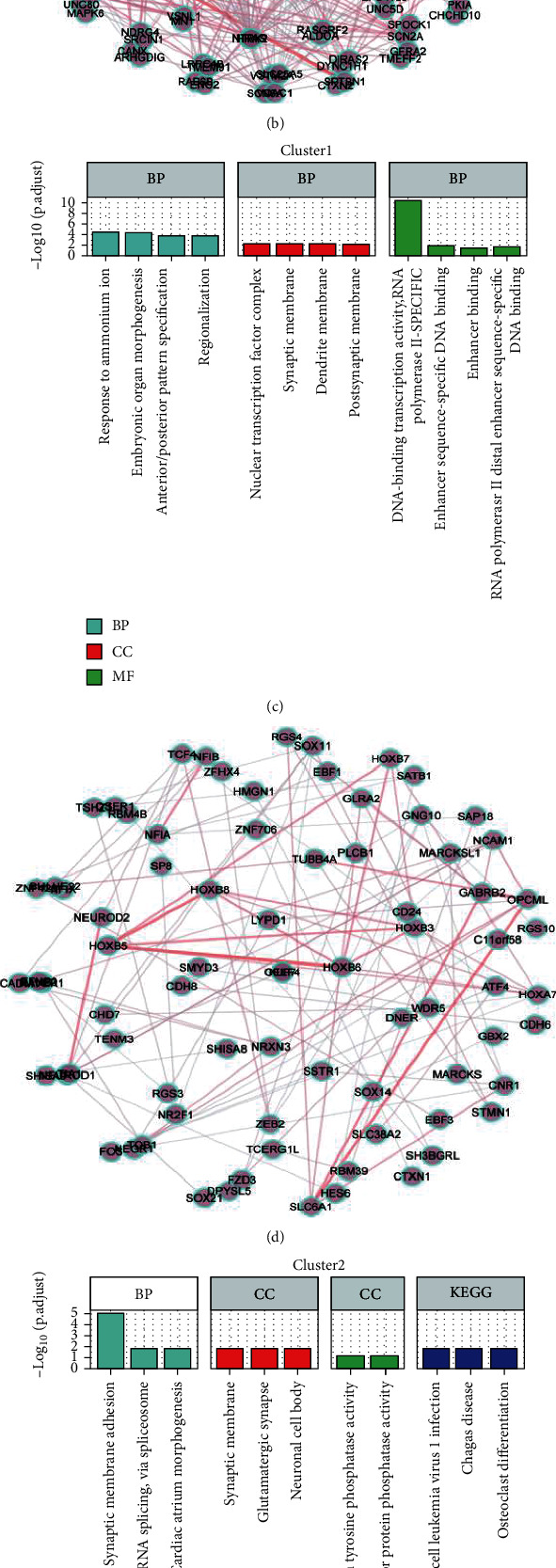
Enrichment analysis of clusters 0-2 and the corresponding PPI network analysis. (a, b) Enrichment analysis and PPI network of the 198 intersecting mRNAs in cluster 0. (c, d) Enrichment analysis of the 129 intersecting mRNAs and PPI network of cluster 1. (e, f) Enrichment analysis of the 54 intersecting mRNAs and PPI network of cluster 2.

Enrichment analysis of the 198 marker genes in cluster 0 showed that the functions of these genes were significantly enriched in the modulation of the chemical synaptic transmission, regulation of transsynaptic signaling, neurotransmitter secretion, axon part, presynapse, postsynaptic specialization, structural constituent of cytoskeleton, calmodulin binding, metal ion transmembrane transporter activity, endocrine and other factor-regulated calcium, reabsorption axon guidance, and prion disease (Figure 6(a)). The PPI network for these genes is shown in Figure 6(b). The enrichment analysis of the 129 marker genes in cluster 1 showed that the functions of these genes were enriched in response to the presence of ammonium ion, embryonic organ morphogenesis, anterior/posterior pattern specification, regionalization, nuclear transcription factor complex, synaptic membrane, dendrite membrane, postsynaptic membrane, DNA-binding transcription activator activity, enhancer sequence-specific DNA binding, enhancer binding, and RNA polymerase II distal enhancer sequence-specific DNA binding (Figure 6(c)). The PPI network for these genes is presented in Figure 6(d).

Enrichment analysis of the 54 marker genes in cluster 2 showed that the functions of these genes were significantly enriched during synaptic membrane adhesion, positive regulation of mRNA splicing-via the spliceosome, cardiac atrium morphogenesis, synaptic membrane, glutamatergic synapse, neuronal cell body, transmembrane receptor protein tyrosine phosphatase activity, human T-cell leukemia virus 1 infection, Chagas disease, and osteoclast differentiation (Figure 6(e)). The PPI network for these genes is presented in Figure 6(f).

**Figure 7:**
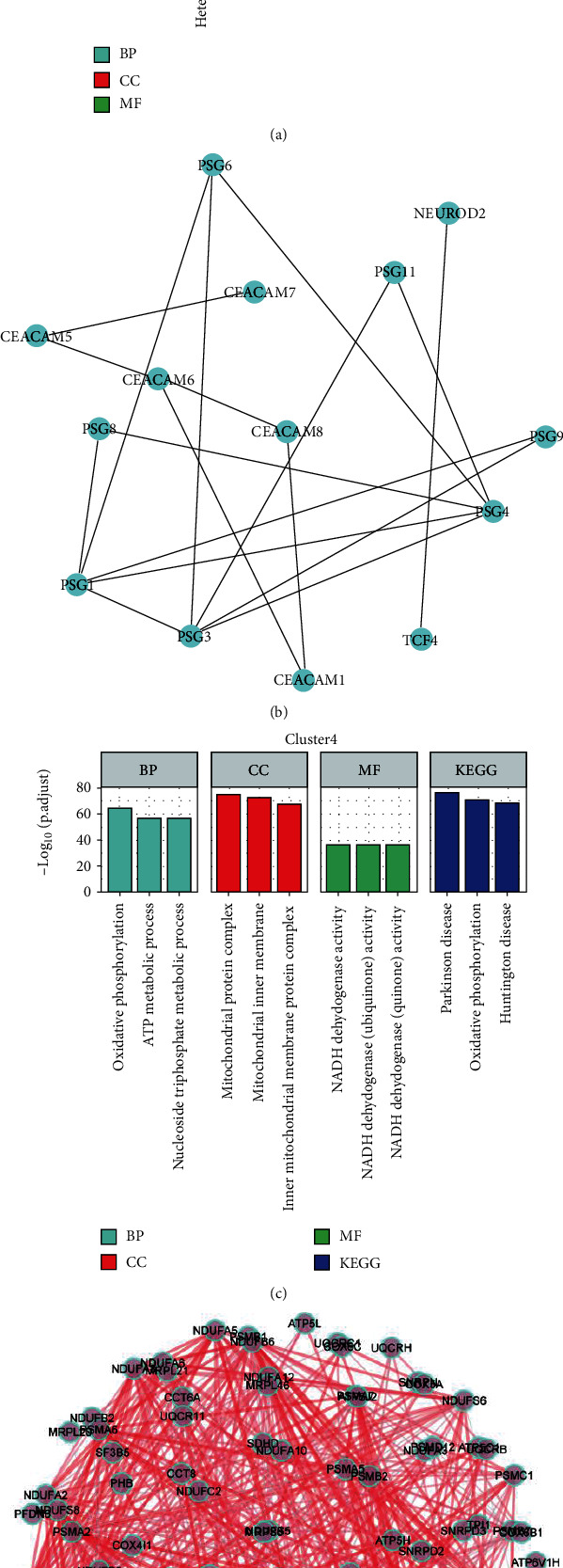
Enrichment analysis of clusters 3 and 4 and their corresponding PPI network analysis. (a, b) Enrichment analysis and PPI network of the 24 intersecting genes in cluster 3. (c, d) Enrichment analysis and PPI network of the 570 intersecting genes in cluster 4.

Enrichment analysis of the 24 marker genes in cluster 3 showed that these genes were significantly enriched during female pregnancy, multicellular organism process, leukocyte migration, and heterophilic cell-cell adhesion via plasma membrane cell adhesion molecules (Figure 7(a)). The PPI network for these genes is presented in Figure 7(b). *Psgs* and *Ceacams* were found to be the core genes of this PPI network.

**Figure 8:**
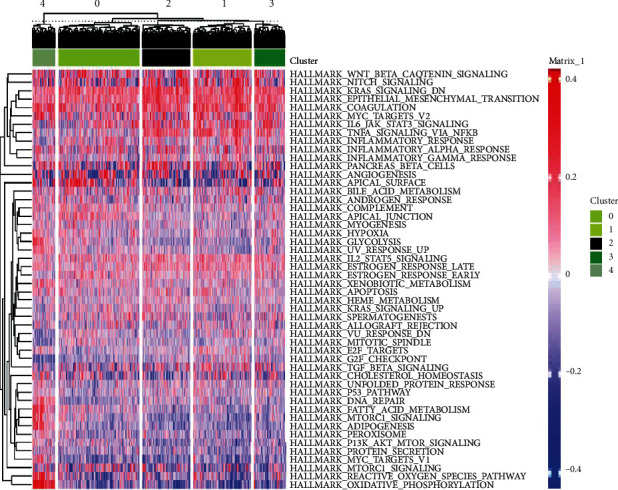
The pathways associated with each cluster as obtained via pathway enrichment analysis.

Enrichment analysis of the 270 marker mRNAs in cluster 4 showed that their functions were significantly enriched in oxidative phosphorylation, ATP metabolic process, nucleoside triphosphate metabolic process, mitochondrial protein complex, mitochondrial inner membrane, inner mitochondrial membrane protein complex, NADH dehydrogenase activity, Parkinson disease, oxidative phosphorylation, and Huntington disease (Figure 7(c)). The PPI network for these genes is shown in Figure 7(d). Finally, GSVA enrichment analysis demonstrated the differential expression of the different pathways in the Reactome database across the different clusters (Figure 8).

### 3.6. Identification of Transcriptional Regulatory Networks Associated with USF2/CEACAM1&5 and KLF6/PSG3&5

**Figure 9:**
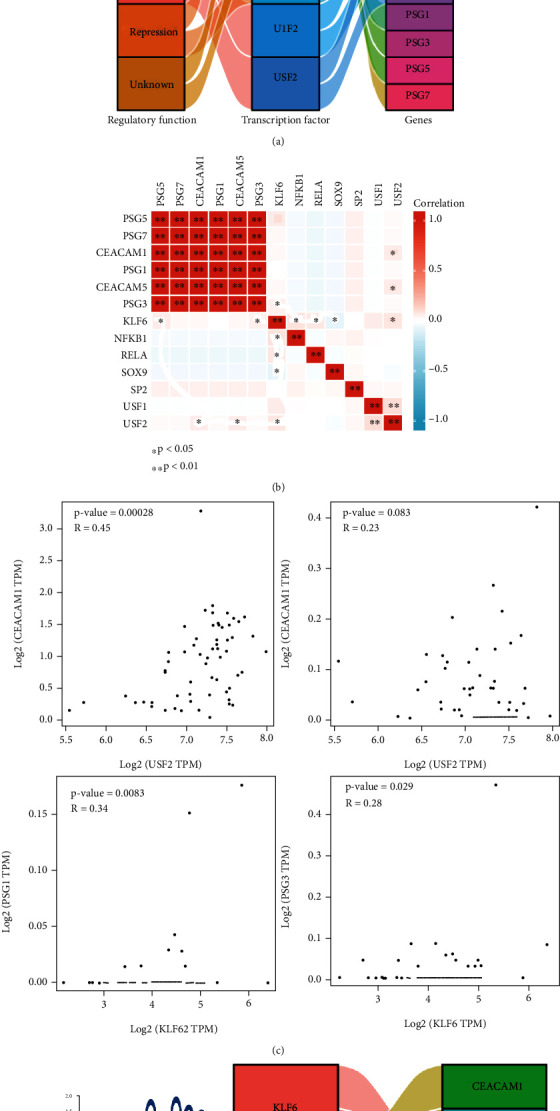
Identification of USF2/CEACAM1&5 and KLF6/PSG3&5 transcriptional regulatory networks. (a) Transcriptional networks of Psgs and Ceacams. (b) Correlation analysis of Psgs, Ceacams, and their potential transcriptional regulators. (c) Correlation analysis based on the GTEX dataset. (d) Motif loci for KLF6 and USF2. (e) Identification of USF2/CEACAM1&5 and KLF6/PSG3&5 transcriptional regulatory networks.

**Figure 10:**
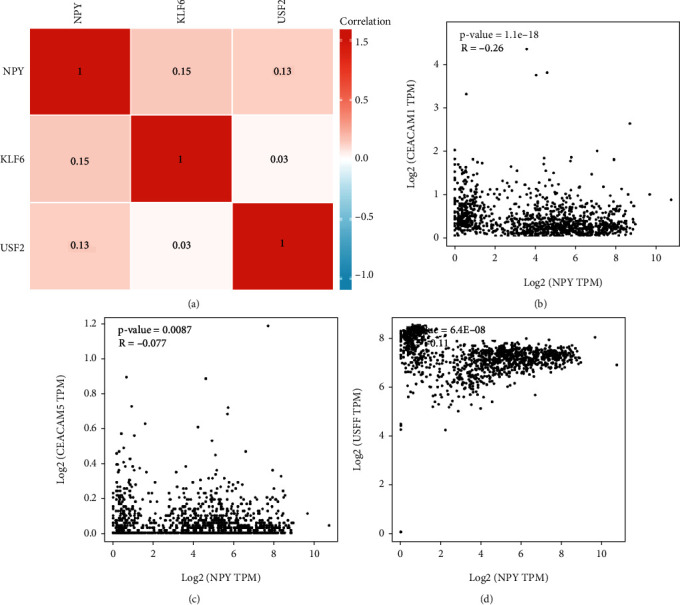
The spinal cord developmental marker NPY and its association with CEACAM1, CEACAM5, and USF2. (a) Correlation analysis of NPY, KLF6, and USF2. (b) Correlation analysis of NPY and CEACAM1. (c) Correlation analysis of NPY and CEACAM5. (d) Correlation analysis of NPY and USF2.

*Psgs* and *Ceacames* were found to be the core genes for cluster 3 in this study. The TRRUST database was then used to find upstream transcriptional regulatory targets for *Psgs* and *Ceacams.* The transcriptional network of PSGs and CEACAMs were shown in Figure 9(a), wherein the upstream transcriptional regulatory targets of *CEACAM1*, *CEACAM5*, *PSG1*, *PSG3*, *PSG5*, and *PSG7* were predicted. *HNF4A*, *IRF1*, *NFKB1*, *RELA*, *SOX9*, *SP2*, *USF1*, and *USF2* were predicted to be transcriptional regulators of *CEACAM1* and *CEACAM5*. *FOXF2*, *KLF6*, and *MYBL2* were predicted to be transcriptional regulators of *PSGs* such as *PSG1*, *PSG3*, *PSG5*, and *PSG7*. Correlation analysis suggested a significant correlation between PSGs and CEACAMs in the GSE108788 dataset (Figure 9(a)). It was also observed that *CEACAM1* and *CEACAM5* were significantly positively correlated with the expression of *USF2* (Figure 9(b)). Similarly, *PSG3* and *PSG5* were significantly positively correlated with the expression of *KLF6* (Figure 9(b)). However, GSE108788-based correlation analysis of scRNA-seq dataset found that the results for FOXF2, HNF4A, IRF1, and MYBL2 did not match the results expected in Figure 9(a). The Genotype-Tissue Expression (GTEx) project is a dataset that reflects the relationship between genetic variants and gene expression in human tissues. In spinal cord samples from the GTEx dataset, *KLF6* expression was found to have a positive correlation with the expression of *PSG1* and *PSG3*, while *USF2* expression was found to have a significant positive correlation with the expression of *CEACAM5*. The binding sites of the motifs of *KLF6* and *USF2* are shown in Figure 9(d) based on the TRRUST database. Based on these results, USF2 was presumed to be a transcriptional regulator of *CEACAM1* and *CEACAM5* while KLF6 is presumed to be a transcriptional regulator of *PSG3* and *PSG5* (Figure 9(e)). The spinal cord developmental marker NPY was positively correlated with *CEACAM1*, *CEACAM5*, and *USF2* (Figures 10(a)–10(d)).

## 4. Discussion

In this study, single-cell analysis was utilized to reveal the heterogeneity and pseudotemporal differentiation trajectory analysis of the neonatal mouse spinal cord neuronal cells. *Psgs* and *Ceacams* were found to be the core genes for cluster 3, where the marker genes in these cell subpopulations characterize the transcriptional profile of mouse spinal cord neuronal cells.

Our results show that the molecular diversity of cell types plays an important role in the development of the mouse spinal cord, similar to previous studies that conducted large-scale molecular profiling [18–21]. The spinal cord nerve cells of mice were divided into five clusters. These results provide new insights into the temporal patterns of gene expression, thus establishing a new framework for the analysis of the mouse spinal cord.

This study provides a clustering map for the clustering of different cells correlated to their function. The cluster 0 marker genes were functionally enriched mainly in synapses with signaling; the marker genes in cluster 1 are mainly enriched in gene transcription; the marker genes in cluster 2 are mainly enriched in transmembrane transport and cellular communication in neuronal cells; the marker genes in cluster 3 are remarkably enriched in female pregnancy and multicellular organism processes; and the marker genes in cluster 4 are mainly related to energy metabolism and some disease pathways including Parkinson's disease, oxidative phosphorylation, and Huntington's disease. These results suggest that the differential enrichment of genes in different cells implies the functional differences in the corresponding regional tissues [22].

The Psg family genes and the Ceacam family genes were found to be the core genes for cluster 3 in this study. Cluster 3 were found to be remarkably enriched in female pregnancy and multicellular organism processes. The pregnancy-specific glycoprotein (PSG) gene is a member of the carcinoembryonic antigen cell adhesion molecule (CEACAM) family genes [23]. Both *Psg* and *Ceacam* family genes play a key role in tumor progression [24]. In mammals, CEACAMs and PSGs are involved in feto-maternal interactions. However, previous studies have not systematically investigated the developmental trajectories of these two groups of genes in humans [25]. In addition, CEACAMs and PSGs have been found play a prominent role in the study of species evolution [25, 26], as the expression of PSG may imply the adaptive evolution of species [27]. Our study revealed that PSGs and CEACAMs are potentially involved in neural development.

KLF6, a zinc-finger transcription factor of the KLF family, is involved in several processes including cell development, differentiation, and regulation [28]. KLF6 was found to be a key transcription factor involved in the central neuro-mediated apoptosis with the function of reducing neurological damage after cerebral hemorrhage [29]. Based on bioinformatic analysis, KLF6 was found to be a potential transcriptional regulator for PSG3 and PSG5 in this study. The upstream stimulatory factors (USFs) USF1 and USF2 all belong to the helix-loop-helix leucine zipper transcription factor family and function as homodimers or heterodimers by binding to the e-box of the target DNA core sequence (5′-CANNTG-3′) [30, 31]. USFs play an important role in stress, the immune response, energy metabolism, and cell development [32–34]. The role of USF2 seems to be more critical than that of USF1 [35, 36]. In this study, USF2 was hypothesized to be a transcriptional regulator of CEACAM1 and CEACAM5.

Neuropeptide Y (NPY) was discovered by Tatemoto et al. in 1982 and was found to be localized in the nervous system; its functional role has been intensively investigated [37]. Previous studies suggested that NPY may function as a neurotransmitter, has a neuromodulatory function, or has neuroendocrine function [36, 38–40]. Although the morphology and distribution of NPY in the adult spinal cord have been reported, the development of NPY during the formation of the human fetal spinal cord and its patterns have not yet been reported in the literature [36, 38, 39]. In this study, the spinal cord developmental marker NPY was found to be positively correlated with CEACAM1, CEACAM5, and USF2.

Advances in single-cell transcriptomics have allowed us to gain groundbreaking insights into the heterogeneous patterns of gene expression over time in the development of spinal nerves. The heterogeneity of neonatal mouse spinal nerve cells was revealed in this study through single-cell analysis. The spinal cord nerve cells of mice were divided into five clusters, giving an initial indication of the complexity of the differential expression of the genes of these cells over time. These results provide new insights into the temporal patterns of gene expression, thus establishing a new framework for the analysis of the mouse spinal cord. Psgs and Ceacams were also defined for the first time in this study as marker mRNAs clustered within the same cell. The upstream transcriptional regulators of the *Psg* and *Ceacam* genes were also identified. In future studies, immunohistochemical experiments, as well as *in vivo* and *in vitro* studies will still be needed to validate our results.

## 5. Conclusions

In summary, we analyzed recently published single-cell datasets and defined five subgroups of marker mRNAs that can be used for future single-cell transcriptome analyses. This study also revealed that *PSGs* and *CEACAMs* are potentially involved in neural development. Identifying these transcriptional regulatory networks will allow us to investigate further the developmental trajectory of mouse neurons and the cell biology of diseases.

## Data Availability

All original data for this study were obtained from the GSE131882 dataset in the GEO database and the Genotype-Tissue Expression (GTEx) project.

## Abbreviations

ANOVA: Analysis of variance
CEACAM: Carcinoembryonic antigen-related cell adhesion molecule
CNS: Central nervous system
GEO: Gene Expression Omnibus
GO: Gene Oncology
GTEX: The Genotype-Tissue Expression
GSVA: Gene set variation analysis
KEGG: Kyoto Encyclopedia of Genes and Genomes
PPI: Protein-protein interaction network
PSG: Pregnancy-specific glycoproteins.
